# Evaluation of Methane Emissions Originating from LNG Ships Based on the Measurements at a Remote Marine Station

**DOI:** 10.1021/acs.est.1c03293

**Published:** 2021-10-08

**Authors:** Tiia Grönholm, Timo Mäkelä, Juha Hatakka, Jukka-Pekka Jalkanen, Joel Kuula, Tuomas Laurila, Lauri Laakso, Jaakko Kukkonen

**Affiliations:** † Finnish Meteorological Institute, Erik Palmenin aukio 1, FI-00560 Helsinki, Finland; ‡ School of Physical and Chemical Sciences, North-West University, PB X6001, Potchefstroom, 2520, Republic of South Africa; § Centre for Atmospheric and Climate Physics Research and Centre for Climate Change Research, University of Hertfordshire, College Lane, Hatfield, AL10 9AB, United Kingdom

**Keywords:** natural gas fuel, shipping emission, methane slip, pollution plume, unburnt methane, maritime transport, climate impact

## Abstract

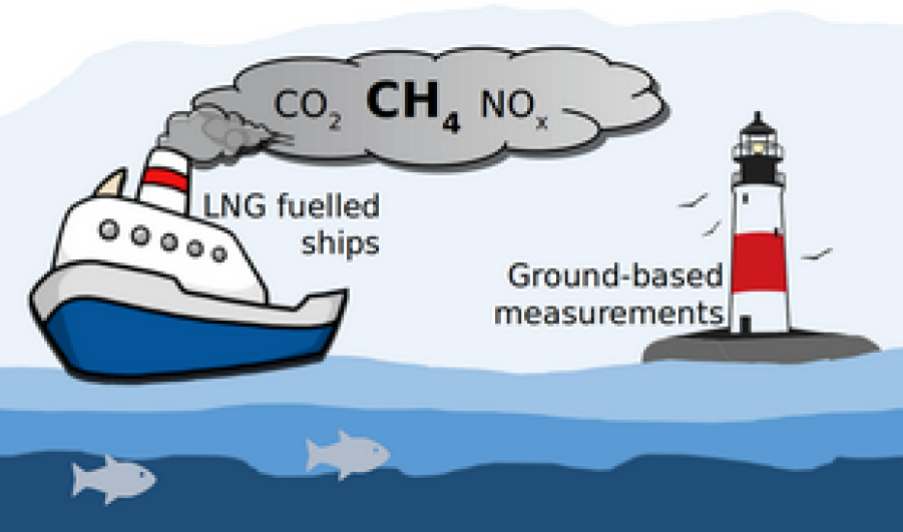

We analyzed pollution plumes originating from ships using liquefied natural gas (LNG) as a fuel. Measurements were performed at a station located on the Utö island in the Baltic Sea during 2015–2021 when vessels passed the station along an adjacent shipping lane and the wind direction allowed the measurements. The ratio of the measured concentration peaks ΔCH_4_/ΔCO_2_ ranged from 1% to 9% and from 0.1% to 0.5% for low and high pressure dual fuel engines, respectively. The ratio of the measured concentration peaks of ΔNO_*x*_/ΔCO_2_ varied between 0.5‰ and 8.7‰, which was not explained by engine type. The results were consistent with previously measured on-board or test-bed values for the corresponding ratios of emissions. While the methane emissions from high pressure dual fuel engines were found to fulfill the goal of reducing the climatic impacts of shipping, the emissions originating from low pressure dual fuel engines were found to be substantially high, with a potential for increased climatic impacts compared with using traditional marine fuels. Taking only the global warming potential into account, we can suggest a limit value for the methane emissions; the ratio of the emissions ΔCH_4_/ΔCO_2_ originating from LNG powered ships should not exceed 1.4%.

## Abstract 2

Remote marine air-quality measurements show significant climate relevant methane emissions from ships using liquefied natural gas as a fuel.

## Introduction

Combustion in ship engines produces a range of primary and secondary pollutants that have important environmental, health, economic, and climatic impacts. The greenhouse gas (GHG) emissions attributed to shipping contain carbon dioxide (CO_2_), methane (CH_4_), and nitrous oxide (N_2_O); these can collectively be evaluated as carbon dioxide equivalent (CO_2_e). The CO_2_e of total global shipping increased 9.6% from 2012 to 2018.^1^ The share of shipping emissions in global anthropogenic CO_2_e emissions was 2.89% in 2018.^1^

In addition to GHG emissions, shipping produces significant emissions of other pollutants like sulfur and nitrogen compounds. In an aim to reduce the environmental impacts, new global standards have been applied since January 1, 2020 for marine fuels. The new limit for fuel sulfur content (FSC) is 0.5%, which implies a significant decrease from the previously allowed maximum FSC of 3.5%. An even stricter regulation of 0.1% FSC has been set in sulfur emission control areas (SECAs) since January 1, 2015. In Europe, these comprise the Baltic and North Seas as well as the English Channel. In complying with the limit values within SECAs, ships are currently mandated to use fuel oil within the FSC limits. Alternatively, vessels may (i) be equipped with abatement systems, commonly SO_*x*_ (sulfur oxides) scrubbers, that decrease sulfur emission in the exhaust to reach the limits or (ii) switch to alternative fuels, including liquefied natural gas (LNG).

The introduction of vessels using LNG as a fuel may be a promising and economically viable solution in the future, especially as it may also comply with NO_*x*_ ECA emission limits.^2−5^ The high energy content of LNG enables a more efficient consumption of fuel, compared to using liquid fuels. LNG is also a clean fuel in most respects. Anderson et al.^6^ performed particle number size distribution and exhaust gas measurements onboard an LNG dual fuel ship. They found that emissions of particles, NO_*x*_, and CO_2_ were clearly lower when using LNG instead of marine fuel oils. Alanen et al.^7^ also observed lower particle emissions from an LNG engine, compared to marine diesel oil (MDO) or marine gas oil (MGO). Moreover, Peng et al.^3^ observed 93%, 97%, and 92% reduction of emissions in particles, black carbon, and NO_*x*_, respectively, when changing from diesel fuel to LNG as a fuel. However, at the same time, the folmaldehyde (HCHO), carbon monoxide (CO), and CH_4_ outflow increased several-fold.^3^

The contribution of unburnt methane should not be neglected when considering the GHG emissions and emission reduction agreements from the shipping traffic. Increased CH_4_ emission results in difficulties with emission reductions of greenhouse gases. Over the period 2012–2018, the CH_4_ emissions of shipping increased 87%, partly due to increased consumption of LNG and the increase in the use of dual-fuel machinery that has higher specific exhaust emissions of CH_4_.^1^ A large scale introduction of LNG powered ships may in the worst case result even in an increased climatic impact. The emission of unburned methane in marine engines (also called as the methane slip) depends on engine load; it is largest for lower loads. Methane slip emissions can impact climate change since methane has 28–34 times higher global warming potential than CO_2_ over a time span of 100 years (GWP100), see e.g., refs (8) and (9). As pointed out by Ushakov et al.,^4^ methane slip has been previously ignored but has recently received more attention.^1^ However, the amount of relevant measurement data is scarce regarding the methane slip;^3^ in addition, the existing data originated mainly from test-bed measurements by engine manufacturers. In previous research, no studies have been presented that have measured and analyzed measurement data for several ships that use LNG-fueled marine diesel engines in their actual operating environments. However, such data are urgently needed to be able to realistically analyze the climatic impacts of the rapidly increasing LNG powered shipping.

The overall aim of this study was to evaluate, quantitatively, the methane emissions originating from a wide range of LNG powered ships. The more specific objectives were (i) to quantify the methane emissions for the LNG ships that have traveled past the Utö island in the Baltic Sea during 2015–2021, (ii) to evaluate the impacts on the methane emissions in terms of the different types of engines of the ships, and (iii) to discuss the importance of methane slip in terms of the climatic impacts and potential future emission limits.

## Materials and Methods

### Engine Types and Methane Slip

Currently, there are three engine types available for marine applications; these are based on different combustion characteristics. According to Ushakov et al.,^4^ they can be grouped as (1) lean burn spark ignited engines, for medium and high speed with a four-stroke cycle (LBSI), producing a power range of 0.5–8 MW; (2a) low pressure dual fuel engines for medium speed with a four-stroke cycle (LPDF) producing 1–18 MW; (2b) low pressure dual fuel engines for low speed with a two-stroke cycle (LPLSDF) producing 5–63 MW; (3a) a high pressure dual fuel engine for medium speed with a four-stroke cycle (HPMSDF) producing 2–18 MW; and (3b) a high pressure dual fuel engine for low speed with a two-stroke cycle (HPLSDF) producing over 2.5 MW. Stenersen and Thonstad^10^ report that in December 2016, approximately 120 LNG vessels were in operation, from which approximately 41% had type 1, 47% type 2a, 3% type 2b, and 8% used type 3 engines.

The spark-ignited and low-pressure dual fuel engines have low NO_*x*_ emissions, making them attractive from the point of view of the Nitrogen Oxide Emission Control Area (NECA) operation, as they are IMO tier III compliant. However, these engines emit unburnt methane, especially at low engine loads. In contrast, the high-pressure engines emit much less methane but are only IMO tier II compliant, which necessitates the use of exhaust gas after-treatment, such as Selective Catalytic Reduction (SCR), to achieve tier III NO_*x*_ reduction. The reduction of NO_*x*_ emissions by using low-pressure dual fuel engines is a tempting feature for ship fleets operating on NECAs, but in the absence of a regulation for the methane slip, methane emissions would be likely to increase.

In an internal combustion engine, the emissions of NO_*x*_ are formed predominantly through a process called thermal NO_*x*_ formation: the diatomic nitrogen present in the combustion air oxidizes at high temperatures and forms nitrogen oxides. The rate of NO_*x*_ formation is in part dependent on the combustion temperature; by reducing the peak combustion temperature, one can therefore achieve a reduction of the formed NO_*x*_ emissions. This is one of the design principles of the LBSI and LPDF engine types. They operate on the basis of the so-called Otto cycle, applying a lean air–fuel mixture ratio, which translates into low thermal loading.

In an LBSI engine, the ignition of the air–fuel mixture is done using a prechamber, in which a spark plug ignites a locally enriched air–fuel mixture. Similarly, in an LPDF engine, the air–fuel mixture is ignited in a prechamber using pilot diesel fuel. The drawback of these engine types is the emitted unburnt methane, or methane slip, which occurs due to two factors. First, the lean combustion and consequent low thermal loading makes the engine susceptible to quenching. Quenching means that the methane cools down rapidly in the combustion chamber, and, as methane requires a relative high temperature to autoignite (>600 °C), it may remain unignited. Methane slip resulting from quenching is particularly intensive at low engine loads as shown, for example, by Ushakov et al.^4^ A secondary cause for methane slip is related to the working principle of the Otto cycle. In an Otto engine, the fuel is injected to the intake manifold, and a homogeneous air–fuel mixture is then formed in the combustion chamber before its ignition. The residence of methane in the combustion chamber before its ignition allows it to be pushed to the crevice volumes of the chamber, for example, to the gasket area between the cylinder head and cylinder liner, during the compression stroke. This may prevent its ignition and thus result in a methane slip.

The methane slip can be considered to be caused by the trade-off of the emissions of NO_*x*_ and CH_4_. An engine, such as the LBSI and LPDF, can be optimized to run with a minimal thermal loading, resulting in low NO_*x*_ emissions. On the other hand, the downside of these types of engines is the emitted methane slip. In contrast, the diesel cycle-based high-pressure engines (HPMSDF and HPLSDF) are less susceptible to quenching, due to their compression ignition-based operation and higher thermal loading. Moreover, the methane slip due to crevice volumes is not an issue, as the methane burns as it is being injected to the combustion chamber; there is no possibility for it to escape to the crevice volumes during the compression stroke. However, high-pressure engines emit NO_*x*_ emissions to the degree that requires them to be equipped with an SCR system to be IMO tier III compliant. As methane emissions—unlike NO_*x*_ emissions—have remained unregulated up to date, it is to be expected that shipping companies consider the use of LNG engines producing low NO_*x*_ emissions.

In the future, an improved design of engine components and engine process control may further reduce the methane slip.^10^ However, the IMO should consider the introduction of methane emission limits as soon as possible, and the regulatory framework should be sufficiently comprehensive. In the current regulatory approach, only emissions originating from ships are considered. From the point of view of shipping emissions, the fossil, bio-, and synthetic methane are equivalent. However, these various methane fuels are substantially different in view of their climatic impacts, if one will also consider the impacts during their production process.

### Measurement Site

**Figure 1:**
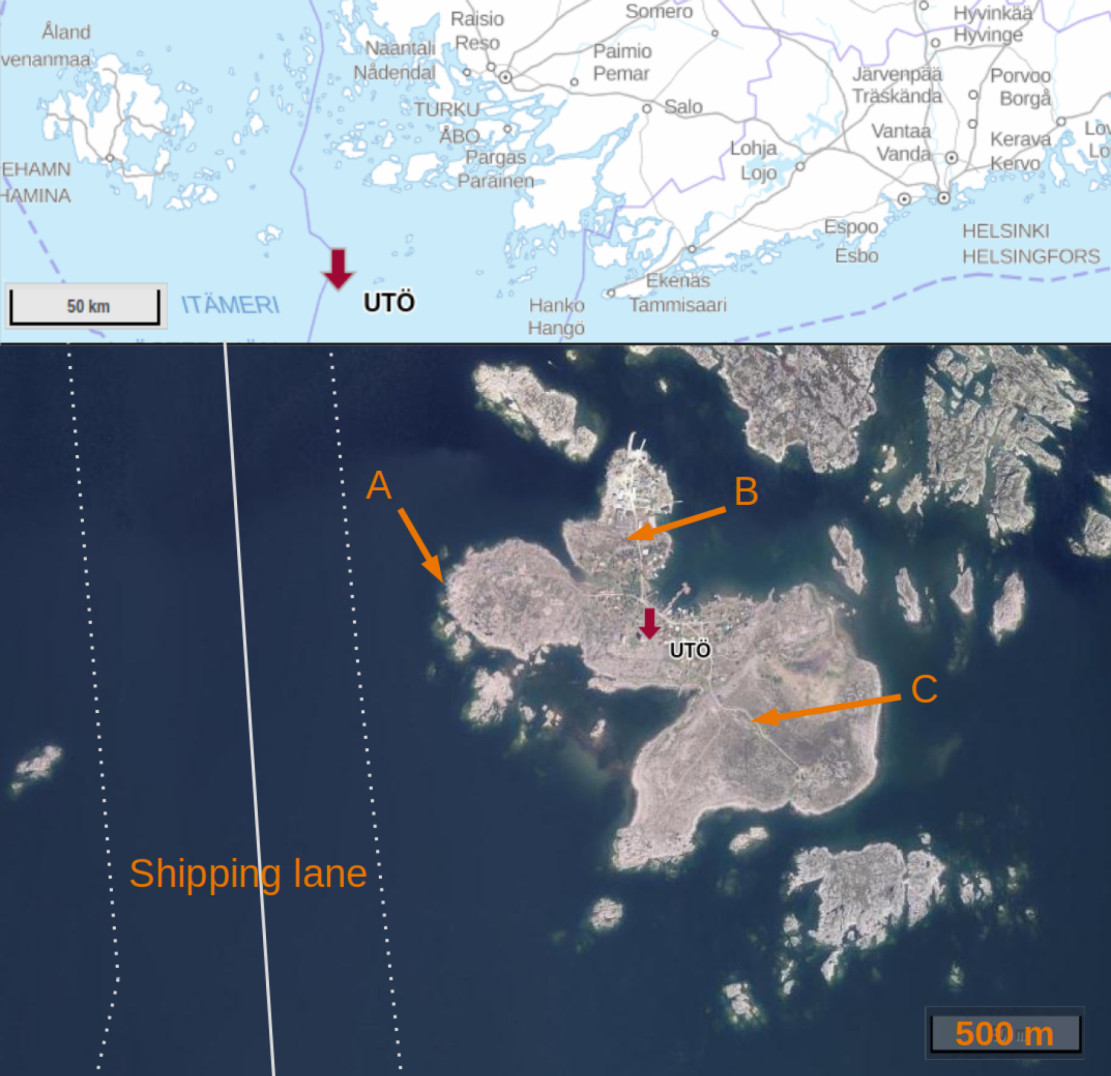
Upper panel, the location of Utö island (red arrow) on the southern coast of Finland. The lower panel presents an aerial photo of Utö island and its immediate surroundings. The arrows A, B, and C show the locations of the sea station, the ICOS station, and the air quality station, respectively. The thin solid white line in the sea area indicates the shipping lane (≈ 600 m from the island), and the lane area (300–1100 m from the island) is marked with dotted lines. Copyright of the map and the aerial photo is owned by National Land Survey of Finland and licensed under a Creative Commons Attribution 4.0 International License.

Utö is an island with an area of 0.81 km^2^ (N59° 47.034′, E21° 22.030′) situated in the Baltic Sea, approximately 90 km south of continental Finland and the closest city Turku. Utö is a bare, rocky island with some low-growing vegetation, mainly consisting of bushes and with a permanent population of less than 50 people. A map showing the location of Utö island at the Finnish southern coastline is given in Figure 1. A densely trafficked shipping lane passes to the west of the island at a distance of about 600 m. Besides the shipping emissions, there are no other significant sources of CH_4_ or CO_2_ in the area.

Meteorological conditions are windy. Monthly wind speed conditions ranged from 5.6 m s^–1^ in July to a maximum of 8.9 m s^–1^ in December with an average of 7.1 m s^–1^ over the year. The prevailing wind direction is southwest; the winds from the south–northwest sector prevail for approximately 60%.^11^ The measurements at Utö Atmosphere and Marine Research Station are described in detail in Laakso et al.^12^

### Experimental Setup

We have used the data measured at the Integrated Carbon Observation System (ICOS) Atmosphere Station and at the Marine Research Station. The measurements at these two stations have been briefly described in the following. For a more detailed description of these measurements, the reader is referred to Laakso et al.^12^ and Kilkki et al.^13^

### The ICOS Atmosphere Station

The atmosphere station of Utö is for long-term high-precision observations of greenhouse gases. The station has been part of the European ICOS atmospheric measurements network since March 2018. The location of the station is shown in Figure 1, marked with the arrow B. The 60 m tall tower is located at a distance of approximately 200 m from the coastline in the northern part of the island. The concentrations of CO_2_ and CH_4_ were measured using a Cavity Ring-Down Spectrometer (CRDS), G2401 (Picarro, Inc., Santa Clara, CA, U.S.A.), together with calibration standards traceable to the scales of WMO CCL (World Meteorological Organization, Central Calibration Laboratory). In addition, the Picarro instrument measured carbon monoxide (CO) and water vapor, the latter of which was used by the analyzer to calculate dry gas concentrations. The analyzer measured each species consecutively within ca. 2.5 s. According to the manufacturer, the precision on a time period of five seconds is better than 1 ppb for CH_4_ and 0.05 ppm for CO_2_. Sampling height was 56 and 64 m with respect to the surrounding terrain and the mean sea level, respectively. The length of the sampling line was 124 m. The time lag caused by the sampling line was about 40 s. At the top of the tower, wind speed and direction were measured with a 3-D sonic anemometer, USA-1 or uSonic-3 Scientific (Metek GmbH, Elmshorn, Germany), which recorded the three wind components and the acoustic temperature at a frequency of 10 Hz.

### The Marine Research Station

The Marine Station is situated approximately at a distance of 500 m to the west of the ICOS station (Figure 1, location A). This station is located at a horizontal distance of a couple of meters from the shore. The station consists of a 9-m-tall mast, which is mounted on a cliff that is approximately at a height of 3 m above the mean sea level. The mast is mainly used for micrometeorological greenhouse gas flux measurements; it contains a Metek USA-1 3D sonic anemometer and an LI-7000 fast-response infrared gas analyzer (Licor Biosciences, Lincoln, NE, USA) for measuring the CO_2_ and H_2_O concentrations. In addition, the Marine Station includes a Thermo Scientific 42i NO–NO_2_–NO_*x*_ analyzer for measuring the concentrations of nitrogen oxides and a Thermo Scientific 43i-TLE analyzer for measuring the SO_2_ concentrations. In addition to the atmospheric observations, a large number of underwater variables are measured at the station, including sea surface dynamics and marine bio-geochemistry.

**Figure 2:**
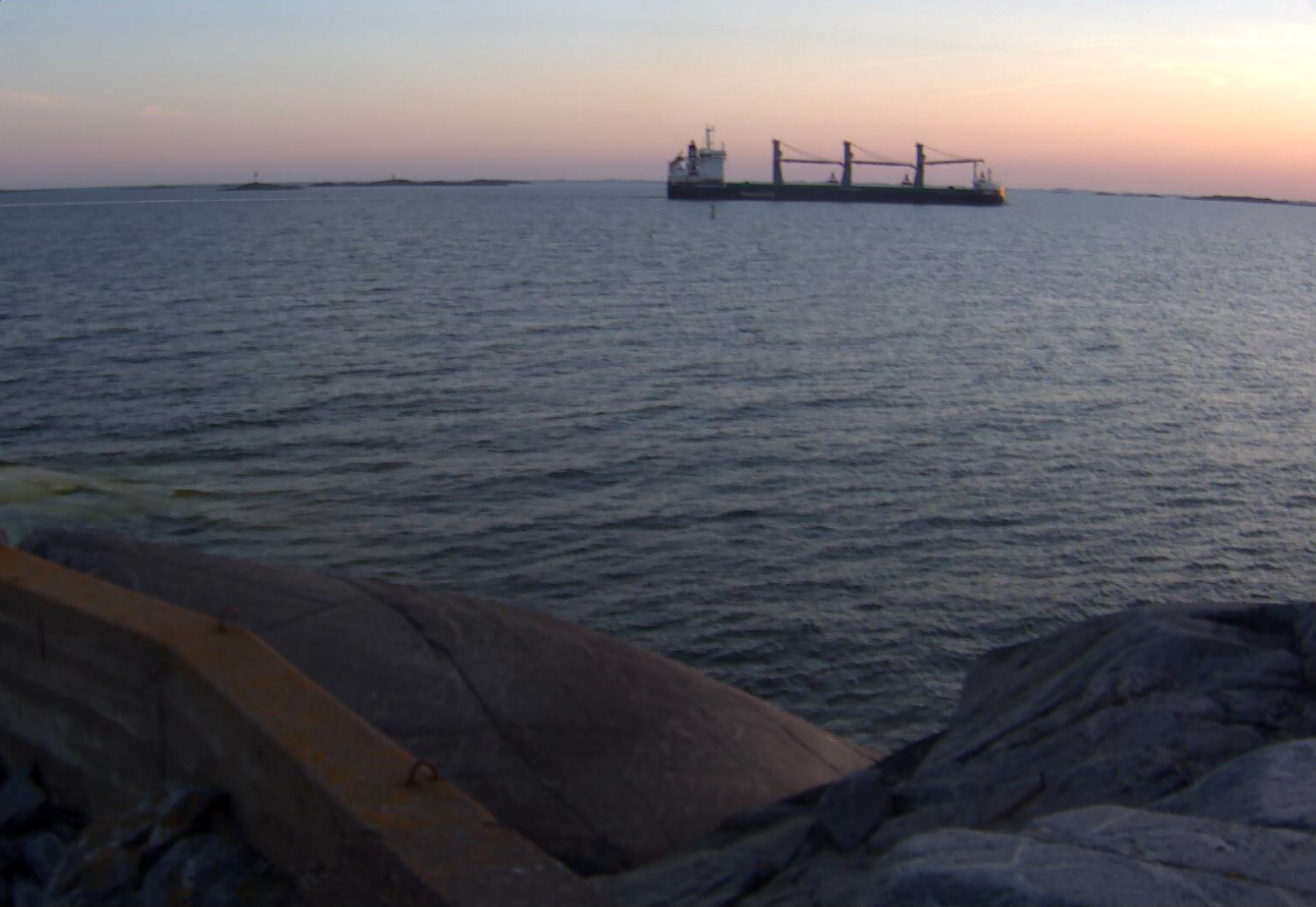
An example of the photo by the camera which was automatically photographing when an AIS-equipped vessel was in the vicinity of Utö island. The photo is taken to the west from the Marine Station. The passing vessel is driving along the shipping lane in a north–south direction approximately 500 m from the island heading to the north.

Marine vessels are identified by an AIS (Automatic Identification System) receiver and a camera setup, which were mounted in the Sea Station. The camera setup was programmed to take a photo every 30 s whenever received AIS-data indicated that a ship was close to the station, as seen in a photo in Figure 2. The received AIS data include, for example, the MMSI-code (Maritime Mobile Service Identity), speed over ground, turning rate, position, and true heading of the ship. This facility enables one to connect the detected pollution plumes with individual passing ships.

### Preprocessing of the Measurement Data

To detect a pollution plume originating from a passing ship, the wind direction has to be in a specific sector from a direction of the shipping lane toward the two applied measurement stations. A further examination of the emissions was started if there was (i) a simultaneous increase of the concentrations of CO_2_ and CH_4_, (ii) the maximum peak value for CH_4_ was larger than 5 ppb, and (iii) the maximum peak of CO_2_ was higher than 0.5 ppm above the corresponding background concentration value. The CH_4_ concentration peaks were easy to detect, due to a temporally fairly constant background. However, if the CO_2_ concentration was close to the background level (no clear peak), the pollutant plume could have originated from a regional scale distance. Such cases were therefore excluded from further analysis.

On the basis of the AIS information on each passing ship, we compiled the type of the engine of that particular ship based on a ship properties database. If the ship used LNG as a fuel, we analyzed the wind direction and both the measured concentrations of CH_4_ and CO_2_. We also subtracted the background concentration from the measured peak concentration data. The auxiliary variables contained in the AIS signals, such as vessel speed and direction, were also archived for such cases. In addition, the relevant measured meteorological data, such as the wind speed and direction, were saved.

An objective of this study was to study the pollutant plumes in terms of the engine types of the LNG ships. We have therefore marked the studied ships using letters (T, P, C, R, F, V, H, and M) in presenting the results. These ships were then divided according to the main engine types, as categorized by Ushakov et al.^4^

## Results and Discussion

### The Passes by of the LNG Fueled Ships

The passes by of LNG fueled ships past the island of Utö have been compiled in Table 1. In total, we were able to observe 38 LNG ship passes by (eight different ships) during the period 2015–2021. The numbers of passes by varied substantially from year to year showing an increasing trend during the most recent years; in particular, in 2020, LNG ships passed the station 20 times. The conditions satisfied the above-mentioned criteria for detecting experimentally the pollution plume a total of 18 times. The wind directions that were favorable for the detection of the plumes ranged from 184° to 331°.

**Table 1:** Numbers of LNG Fueled Vessels That Have Passed the Island of Utö Shown Separately for Each Year during 2015–2021^a^

vessel	T	P	C	R	F	V	H	M	
produced	2014	2016	2017	2018	2015^b^	2018	2018	2020	
ship type	other	other	tanker	tanker	tanker	cargo	cargo	passenger	
engine type	2	2	2	2	2	3	3	2	
year when passed Utö									sum
2015	1								1
2016	1								1
2017	1	2							3
2018	2	2							4
2019	2	1	4						7
2020	1	1	2	2	5	2	2	5	20
2021	1	1							2
sum	9	7	6	2	5	2	2	5	38

a Individual vessels are referred to using letters (T, P, C, R, F, V, H, and M). The types of ships, the production year, and their engine types have also been presented. For example, vessel T passed Utö nine times during Jan 2015 to Feb 2021.

b The engine production year.

**Figure 3:**
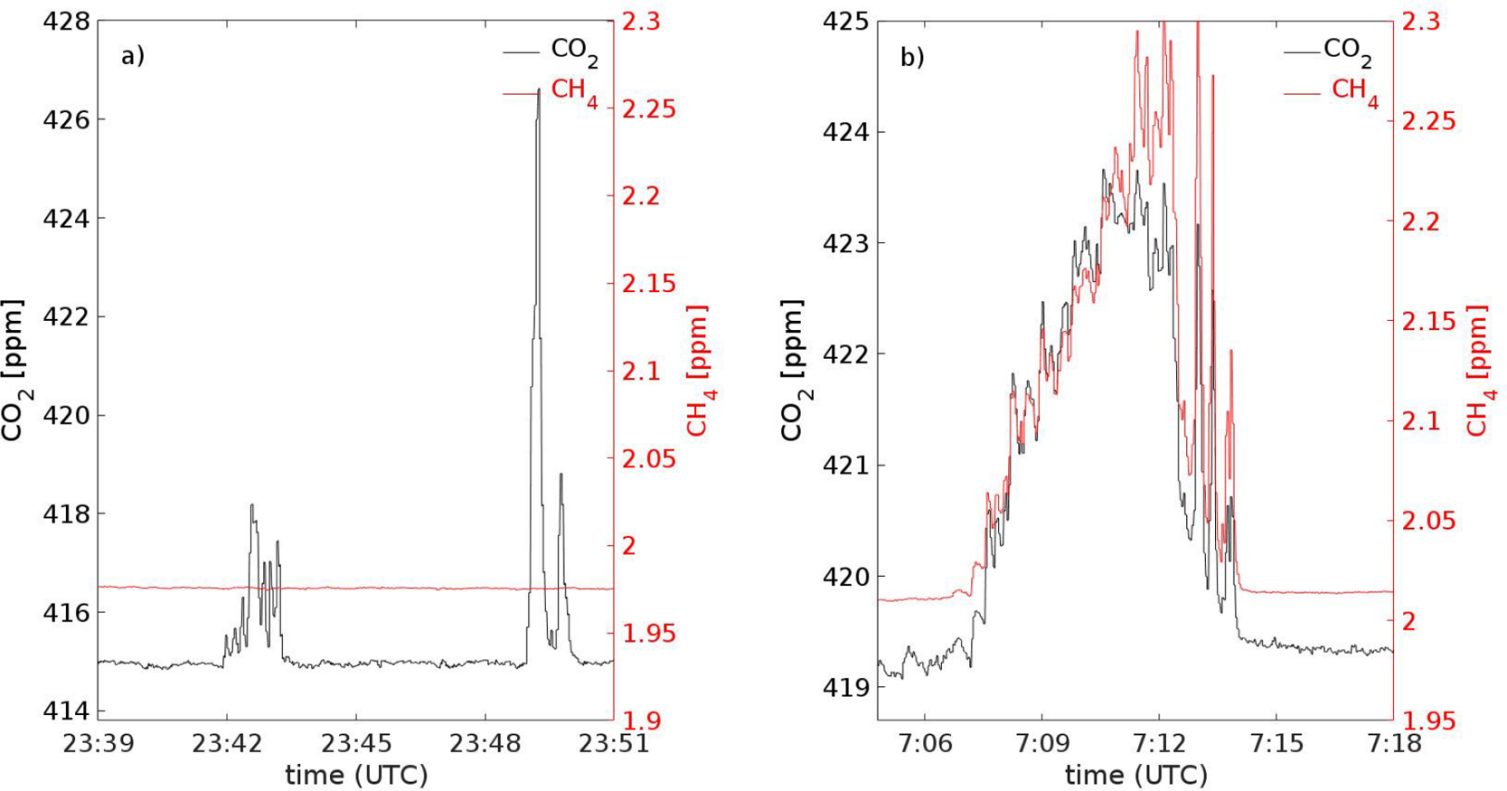
Examples from two different days presenting the measured concentrations of CO_2_ and CH_4_ at the ICOS station on the island of Utö during the passes by of ships. The panels show measured concentrations for the plumes from two passing cargo ships using a diesel engine on November 5, 2020 (panel a) and one ship using an LNG fueled engine (type 2a) on October 5, 2020 (panel b). The CO_2_ concentrations are shown by the black curves, using the left-hand-side axis, and the CH_4_ concentrations by the red curves, using the right-hand-side axis. Note that values depend also on the plume dispersion, and the absolute values are not fully comparable.

Characteristic measured concentration variations at the ICOS station for two passes by of ships have been illustrated in Figure 3. If the vessel is equipped with a diesel engine (panel a), the concentration curves contain a distinct peak of CO_2_ and there is no increase in the concentration of CH_4_. However, for a pass by of a vessel using LNG as a fuel (panel b), both the concentrations of CO_2_ and CH_4_ show a clear increase. Panel b corresponds to a pass by of vessel M, which was traveling in the northern direction in a prevailing strong wind (wind speed 12 m/s) from the southerly–southwesterly direction. In this particular case, the pollutant plume reached the station already before the vessel had passed the island. The measured concentrations of CO_2_ and CH_4_ at the ICOS station were increasing slowly for a period of approximately ∼5 min. After the ship had passed the station, increased turbulence by the wake of this large cruise ship caused an intensive fluctuation of the measured concentrations. Naturally, the absolute values and total measurement time depend on how well the plume reaches measurement devices at Utö island, and therefore they cannot be directly compared between different passes by.

### The Ratios of the Concentrations of Methane and Carbon Dioxide in the Pollution Plumes

First, the background concentrations were subtracted from the measured concentrations; this results in the concentrations attributed to the local ship emissions (increment values, marked with ΔCH_4_, ΔCO_2_, or ΔNO_*x*_). Second, we have plotted ΔCH_4_ as a function of ΔCO_2_. A simple linear regression equation without an intercept was used, ΔCH_4_ = *k* × (ΔCO_2_), where *k* is the slope of the linear regression. Clearly, *k* is also the ratio of the gas concentrations.

The measurements during the passes by of the LNG fueled ships have been summarized in Table 2. The table summarizes the dates of the passes by, the engine types of the passing ships, the maximum peak height values, details on how the passes by occurred, and selected meteorological parameters. The table includes also the ΔCH_4_/ΔCO_2_ ratios calculated from measurements at the ICOS station, the adjusted coefficient of determination of the corresponding numerical fits (*R̅*^2^), and also the ΔNO_*x*_/ΔCO_2_ ratios derived from observations at the Sea Station.

**Table 2:** A Summary of the Measurements of the Methane and Carbon Dioxide Emissions Attributed to LNG Fueled Vessels That Have Passed the Island of Uto^a^

1	2	3	4	5	6	7	8	9	10	11	12	13	14	15
date	engine	ship	ΔCH_4_/ ΔCO_2_ [%]	*R̅*^2^	ΔCH_4max_ [ppb]	ΔCO_2max_ [ppm]	ΔCH_4max_/ ΔCO_2max_ [%]	detect. time [s]	lag [min]	speed mean/min/max [kn]	direction [deg]	WS [ms^–1^]	WD [deg]	ΔNO_*x*_/ ΔCO_2_ [‰]
2015–8–26	2a	T	3.0	0.98	77	2.4	3.1	55	+6	14.3/14.1/14.5 cons	177	9.6	246	no data
2017–11–06	2a	T	5.1	0.99	256	4.5	5.7	86	–1	7.7/7.5/7.9 cons	357	7.7	216	0.5
2018–3–18	2a	T	9.3	0.98	212	2.3	9.3	54	+3	10.4/10.3/10.6 cons	171	9.1	282	1.8
2019–01–15	2a	P	1.5	0.96	12	0.7	1.7	89	+10	13.2/13.1/13.2 cons	20	9.6	331	4.5
2019–12–4	2a	C	1.9	0.85	20	1.0	2.0	48	+3	13.6/13.3/13.9 acc	353	9.5	278	2.3
2019–12–5	2a	C	2.7	0.98	29	1.0	2.8	115	+4	12.9/12.7/13.1 acc	174	13.9	241	3.9
2020–9–13	2a	C	2.1	0.96	27	1.2	2.2	20	+3	13.1/12.9/13.4 acc	174	11.1	255	3.8
2020–2–11	2a	R	4.2	0.87	23	0.7	3.6	48	0	10.5/10.2/10.7 acc	349	11.6	234	4.7
2020–2–11	2a	R	2.8	0.99	44	1.6	2.7	153	+5	11.4/10.6/11.9 acc	179	9.8	240	6.3
2020–3–1	2a	F	3.4	0.97	47	1.3	3.6	125	+3	11.8/11.7/11.9 acc	173	12.3	223	6.0
2020–7–19	2a	F	3.0	0.97	94	3.0	3.1	85	+2	12.9/12.7/13 cons	173	4.9	227	7.3
2020–9–18	2a	F	1.2	0.84	38	3.0	1.3	70	+3	12.5/12.4/12.6 cons	354	6.2	263	5.4
2020–9–20	2a	F	1.9	0.97	21	1.0	2.1	128	+6	10.4/9.6/11.5 dec	152	8.7	226	5.7
2020–10–5	2a	M	3.8	1.00	157	4.1	3.8	268	–9	19.3/19.0/19.6 dec	10	12.0	185	1.3
2020–11–6	2a	M	2.5	0.99	123	4.9	2.5	161	+3	20.7/20.6/20.8 cons	174	8.0	266	6.1
2020–11–8	2a	M	6.4	0.98	264	4.1	6.5	169	+8	18.8/18.7/18.9 cons	6	5.7	304	2.6
2020–6–6	3	H	0.5	0.91	7	1.1	0.6	570	+15	8.4/10.5/7.8 dec	165	10.1	186	NaN
2020–6–26	3	V	0.1	0.92	24	21.3	0.1	59	–1	12.0/10.5/13.1 acc	355	4.8	187	8.7

a The columns are (1) the date on which the vessel has passed the island and the pollutant plume was detected; (2) engine type of the passing ship, classified as described in the main text; (3) ship code used in this study for an identification of the ships; (4) the ratio ΔCH_4_/ΔCO_2_ concentrations calculated from the slope of a linear fit; (5) the coefficient of determination *R̅*^2^ value of the fit; (6) maximum concentration peak height of CH_4_ (max(CH_4_) – background(CH_4_)); (7) maximum concentration peak height of CO_2_; (8) the ratio ΔCH_4_/ΔCO_2_ calculated from the maximum peak height values (columns 6 and 7); (9) duration of the measurement; (10) time lag between the ship passing the island and the plume detection (minus sign indicates that the plume was detected before the ship passed the island); (11) mean, minimum, and maximum speed of the vessel and a description of whether the speed was constant (cons), accelerating (acc), or decelerating (dec); (12) the travel direction of the vessel; (13) local wind speed; (14) local wind direction; (15) the ratio of ΔNO_*x*_/ΔCO_2_ calculated from the slope of the linear fit.

*R̅*^2^ describes the proportion of variance in the dependent variable, which can be explained by the independent variable, also taking into account the number of variables in the model. *R̅*^2^ ranged from 0.84 to 1.00 for the considered individual cases. This indicates that both concentration signals most likely originated from the same local source. Both CO_2_ and CH_4_ can be considered to be chemically inert in the short transport times considered in this study; both gases are therefore dispersed and transported in the atmosphere in almost the same manner. The ratio of ΔCH_4_ and ΔCO_2_ in the ship plumes is therefore directly indicative of the amount of methane slip in the emissions.

The pollutant plumes were detected at the measurement station for a substantial time period, the detection period ranged from 20 to 570 s (Table 2). In some cases, the plumes were detected already before the ship had passed the station, typically in the case of a tailwind. In some other cases, the plume was detected after the ship had already passed the station, typically in the case of a headwind. The first detection time ranged from −9 min to +15 min with respect to the pass by of the ship, defined according to the shortest distance between the ship and the station. The minus sign indicates that the plume was detected before the ship passed the island. The variation in the first detection time is influenced by meteorology, the vessel’s speed and direction, and the height of the exhaust funnels. For example, the release height of the exhausts for vessel M was approximately 65 m, which is comparable to the height of the measurement tower.

The rows in the table are organized so that the separate visits by the same vessel are ordered in time. The ΔCH_4_/ΔCO_2_ values calculated based on the linear fit ranged between 0.1% and 9.3%. There were five vessels, which had traveled past the island of Utö more than once under the conditions for which the plume detection in the stations was possible: the ships T, C, R, F, and M. For the ship T, the ΔCH_4_/ΔCO_2_ ranged 3.0–9.3%; for C, 1.9–2.7%; for R, 1.9–2.7%; for F, 1.9–3.4%; and for M, 2.5–6.4%. The ships P, H, and V have been observable only once with a ΔCH_4_/ΔCO_2_ of 1.5%, 0.5%, and 0.1%, respectively. The lowest values for the CH_4_-slip were observed for the ships H and V, both of which were equipped with a class 3 type engine. Plumes emitted by the ships M and T had the highest ΔCH_4_/ΔCO_2_ ratios. The rest of the vessels, C, R, F, and P, had similar values for ΔCH_4_/ΔCO_2_.

In addition to the type of LNG engine, also the engine load could potentially influence our results. We investigated this possibility by correlating the ratio ΔCH_4_/ΔCO_2_ with the speed of the vessel and the wind vector; no statistically significant correlation was found. However, we did not have information on the amount of cargo onboard the detected ships; clearly, this is one of the key factors in view of the engine load.

The ratio ΔNO_*x*_/ΔCO_2_ varied from 0.5‰ to 8.7‰; the average value was approximately 4‰. This ratio was not clearly dependent on the type of the vessel engine. These ratios were similar to the corresponding ratios measured by Stenersen and Thonstad.^10^

### The Methane Slip Values in Terms of Ships and Engine Types

**Figure 4:**
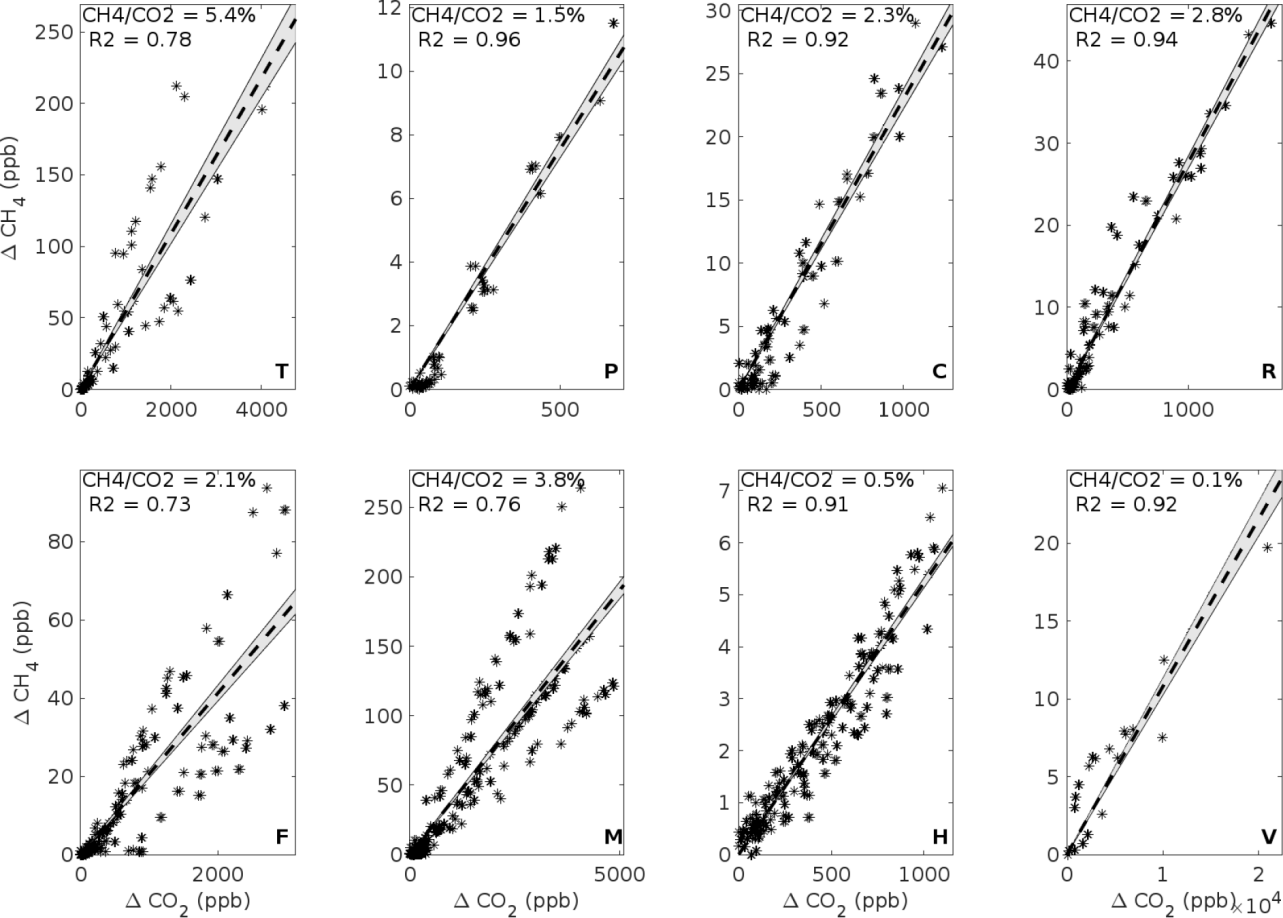
Measured methane concentrations as a function of the measured carbon dioxide concentrations, presented separately for all the passes by of each of the considered ships. The bold letter in the lower right-hand-side corner of each panel indicates the vessel. The dashed line is a linear fit based on all the data points for each vessel; the gray area shows the confidence interval for the fit. The slope of the fit is the ratio of the concentrations of ΔCH_4_ and ΔCO_2_; the value of this ratio is written on the top of each panel.

The same analysis is performed for all plume data we have from passing LNG ships and are shown in Figure 4. By combining all data points from individual passes by, we can get the average ratio (1.5% to 5.4%) for ΔCH_4_/ΔCO_2_. As already shown earlier, the vessels P, H, and V include only one pass by; R, two; T, C, and M, three; and F, four. In particular, it is possible to see that the data from three passes by of vessel M deviate clearly from each other depending probably on the engine load (the values for different slopes presented in Table 2). However, *R̅*^2^ is higher than 0.72 for all of these vessels, and the remaining unexplained variation does not affect the conclusions made based on this data.

**Figure 5:**
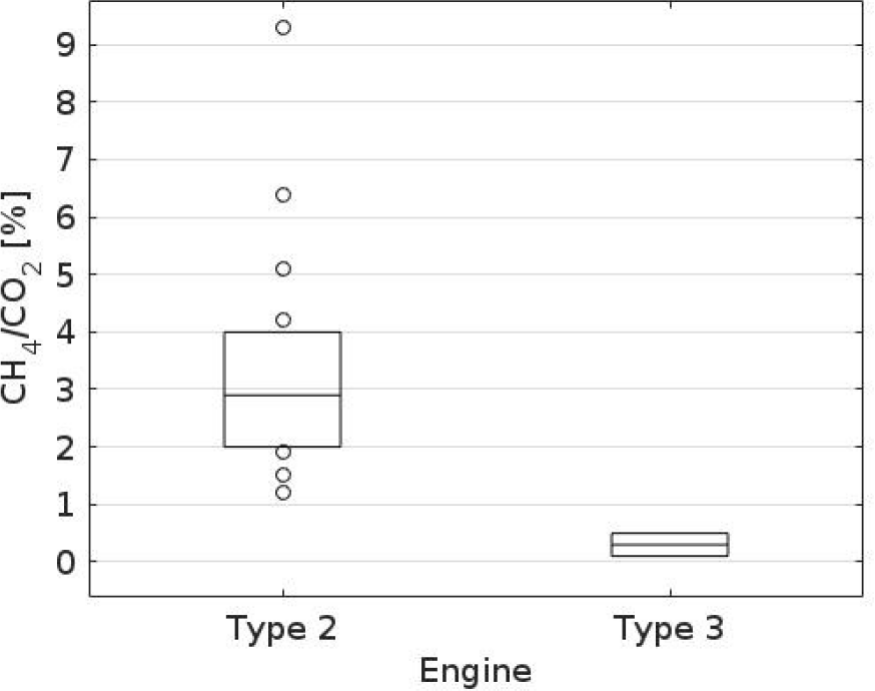
A box plot of all measurements of the ratio ΔCH_4_/ΔCO_2_, presented separately for the engine types of the LNG fueled vessels. The line inside the box indicates the median value. The edges of the box represent the 25th and 75th percentiles, and the open circles show the individual measurements, which were outside the interquartile range. The numbers of values (*N*) for the type 2 engine and the type 3 engine were 16 and 2, respectively.

Figures 4 and 5 present the ratios ΔCH_4_/ΔCO_2_ for all the considered vessels. The data have been presented separately for all the passes by of each specific ship in Figure 4, and for all the passes by of ships having a specific engine type in Figure 5. Clearly, the ships equipped with type 2a engines release more CH_4_ into atmosphere, compared with the corresponding amounts for the type 3 engines. The ratio ΔCH_4_/ΔCO_2_ for type 3 engines was clearly below 1%, ranging from 0.1% to 0.5%. For 2a-type engines, the median value for ΔCH_4_/ΔCO_2_ was approximately 3%, with a range of 1–9%.

The results for 2a-type engines are consistent with the findings of Lehtoranta et al.,^14^ who performed gaseous emission measurements in a laboratory by using Wärtsilä Vasa 4R32, a four-cylinder medium-speed 4-stroke engine (type 2a), which was retrofitted to enable the use of natural gas in dual fuel mode. Lehtoranta et al.^14^ used engine loads of 40% and 75%, and according to their measurements, ΔCH_4_/ΔCO_2_ was ∼8% and ∼4%, respectively. Note that to convert from parts per million by mass to parts per million by volume, the molecular mass of the compounds should be taken into account.

Peng et al.^3^ used a four-stroke, nine-cylinder dual fuel test vessel and observed emissions on-board. According to that study, the ratio ΔCH_4_/ΔCO_2_ with 25%, 50%, 75%, and 100% engine loads was approximately 11%, 6%, 3%, and 2%, respectively. Ushakov et al.^4^ reported emission factors for marine gas engines based on their onboard and testbed emission measurements. For type 2 engines, ΔCH_4_/ΔCO_2_ calculated from the emission factors was ∼4%, and the same for type 1 engines was ∼2%. They did not report any numbers for type 3 engines but claimed that in practice there is no CH_4_-slip. Anderson et al.^6^ performed on-board measurements on a cruise ferry equipped with Wärtsilä 8L 50 DF dual fuel type 1 engine. They measured the amount of total hydrocarbons as a methane equivalent; their results show somewhat lower values, ∼0.7–2.4% for the ratio ΔCH_4_/ΔCO_2_.

### Implications of Methane Slip for Climate Change

When the global warming potential (GWP) of CO_2_ is normalized as 1, for CH_4_ over 100 years the corresponding potential is 28–36.^9^ The CO_2_ emission attributed to LNG fueled ships was found to be 20–30% lower than that to ships equipped with diesel engines.^15^ In the following, we assume that the GWPs for CO_2_ and CH_4_ are 1 and 30, respectively, over 100 years (GWP_100_(CO_2_) = 1; GWP_100_(CH_4_) = 30). We also assume that the normalized CO_2_ emissions originating from vessels equipped with diesel and LNG engines are 1 and 0.7, respectively, (*c*_di_(CO_2_) = 1; *c*_LNG_(CO_2_) = 0.7). In addition, we assume that the emission of CH_4_ attributed to a diesel engine is negligible, (*c*_di_(CH_4_) = 0).

For the sake of a policy, we require that the GWP of diesel and LNG engines be equal. This yields the equation
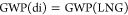
1Taking into account the GWPs attributed to the emission of both CO_2_ and CH_4_, for diesel and LNG engines, eq 1 can be written as
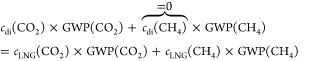
2Arranging eq 2 yields
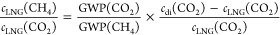
3which, according to the above-mentioned assumptions, has a numerical value
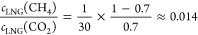
4The implication of this computation is that the ratio ΔCH_4_/ΔCO_2_ in the emissions originating from LNG vessels should be equal to or below 1.4%, if we require that the climatic forcing for 100 years caused by shipping fueled by LNG should be on the same order of or lower than the corresponding forcing using diesel engines. According to the present study, the considered vessels equipped with type 3 engines satisfied this criterion, but none of the vessels equipped with 2a-type engines complied with the criterion.

If the forecasting period would be substantially shorter, for example, 20 years, the GWP for CH_4_ would be ∼85 times higher than that for CO_2_; in that case, the threshold value for ΔCH_4_/ΔCO_2_ would be substantially lower, 0.5%. Even with using this shorter forecasting period, type 3 engines considered in this study would satisfy the criterion. The method that is already presently used in sulfur monitoring, measuring the ratio SO_2_/CO_2_, would be applicable also for monitoring the CH_4_ emissions. This could be implemented by setting up a monitoring network near shipping lanes, by on-board measurements and using other remote monitoring techniques, such as concentration measurements by unmanned aerial vehicles, e.g., drones.

No emission directives or standards are currently in place, which would directly regulate the methane slip for marine LNG engines, e.g., ref (4). In our view, such regulations should be urgently prepared, to mitigate the climatic impacts related to the methane slip of the LNG powered shipping. Such regulations should ideally address the functioning of the marine engines, their emissions including the methane slip, and the environmental and climatic effects of the production and distribution chain of the fuel. On-board measurements of airborne pollution also should be extended to include all the relevant pollutants, in particular, the methane slip of LNG fuelled ships.^10^

In formulating the policies for controlling the CH_4_ emissions attributed to LNG powered ships, one will need to take into account the impacts of emissions to air (also including black carbon) and discharges to sea, especially on human health and marine environments. The cost efficiency of various solutions also needs to be considered. For example, reducing the sulfur emissions is clearly important for human health; however, this could result in a reduction of the formation of aerosol particles, which will have a climatic cooling effect.^16^

Thus, fact-based decision making regulating the shipping emissions should be based on a number of different aspects including impacts of direct emissions of pollutants on the environment and air quality, impacts of fuel production on the climate, air quality and biodiversity, economical costs, security of supply, and climate change. Proper analysis should also include a clear division between the local impacts (e.g., regional air quality) and global, existential crises like climate change and biodiversity loss.
